# Massachusetts Dental Society

**Published:** 1900-02

**Authors:** Edwin E. Davis

**Affiliations:** Boston, Mass.


(To be continued.)

---

### MASSACHUSETTS DENTAL SOCIETY.

(Continued from Vol. XXI., page 58.)

### A SEVERE CASE OF ALVEOLAR ABSCESS WITH NECROSIS.

BY EDWIN E. DAVIS, A.B., D.D.S., BOSTON, MASS.

Mr. President and Members of the Massachusetts Dental Society,—In presenting this paper, I do not presume to give you anything especially new or startling, either as regards the case itself or its treatment and cure.

I do not doubt that many of you have had cases equally severe and cases equally as interesting; yet, since we seldom find among the dangerous cases of alveolar abscess two parallel in their individual characteristics, I shall hope to furnish enough of interest in this to merit your attention for a few moments.

This case is one which came to me about two years ago from the

Emergency Hospital, having been treated there for a week unsuccessfully, in fact, had been growing worse all the time, and, indeed, they had not even located the origin and cause of the trouble. I do not say this with any spirit of criticism, for I think, as a rule, those in charge of the Emergency Hospital are very circumspect and careful.

I will give you as good a description of it as I can. Mr. B. came to my office with the right side of his face well bandaged, looking very pale and sickly, and, indeed, seemed like a very sick man. He removed the bandage, and asked me what I thought the trouble was. I seated him in my chair and observed carefully.

Ordinarily, he wore a full beard, but from the lower part of the right cheek and underneath the jaw the beard had fallen off almost entirely, revealing a badly swollen surface extending from the chin back to the angle of the jaw, and from a point opposite the upper molar teeth down under the jaw and on to the neck. This surface was lumpy and mottled red and purple, almost black in parts, and possessing a peculiar granular feeling as I passed my finger lightly over it.

Reddish, watery pus was discharging from six different openings, and the flow increased upon the slightest pressure. The remaining hairs of the beard on and near this surface came out upon the slightest tension, seeming to start from a point a quarter of an inch below.

I examined the teeth, and discovered on the right side a wisdom-tooth in good condition and a twelfth-year molar heavily filled with amalgam, much discolored, but to my surprise firm, and showing no soreness upon tapping it lightly with my instrument.

I attributed the whole trouble to this tooth, however, and so informed my patient. I asked him some questions, and he gave me the history of the case.

He had noticed about three weeks before a slight swelling just under the jaw on the right side, possessing some soreness, but not sufficient to cause him discomfort. The next day it had increased and began to pain him, and on the third day was so bad that he looked the object of pity that he was, for the pain had become intense. He had experienced several chills and felt sick all over. He gave up his position and went to the Emergency Hospital for treatment. They gave him a hasty examination, omitting, however, to look at his teeth, poulticed his face, and told him to come back in a day or two if no better. His condition grew worse, and with the increased swelling the affair broke out under the jaw and discharged copiously. As he seemed no better the following day, he returned to the hospital, and they told him he would have to take ether, and then they would make an incision and scrape the jawbone, after which they thought he would improve.

After a day or two, during which he was trying to make up his mind to go through the operation, he was advised by a friend, whom I had formerly treated, to come to me. Now, in the treatment of this case, if there are any features which stand out prominently, or in any way render it exceptional, they are two in number,—viz., the direct simplicity of the treatment and the completeness of the cure.

Adjusting the rubber dam to the tooth, I removed all the filling and found that it extended deeply into the body of the tooth, even to the pulp-chamber, where I found a peculiar mass of black, powdery substance. I found no pus, but the odor was sufficiently strong to convince me of the correctness of my diagnosis. Washing the cavity with hydrogen-dioxide and then drying it with alcohol, I easily discovered the nerve-canals; that in the posterior roots large and easily defined, and those in the anterior roots contracted and somewhat tortuous. By careful manipulation the broach was carried to the end of each canal, the apical foramen of each was apparently not enlarged, as the broach did not pass beyond, but brought up solid against it. The canals were thoroughly cleansed with dilute formalin, a small twist of cotton was carried into each and left there, some cotton packed lightly over all, and patient dismissed to come again in two days. I saw the case two days after, and was surprised to find so great an improvement. The discharge had greatly decreased and much of the swelling gone. Very little pain and only slight soreness to the touch. The cotton dressing was removed and the interior of the tooth found to be in a much better condition; very little odor and no moisture. The tooth was dressed as before. Into each of the openings on the face I passed a probe, carrying cotton wet with aromatic sulphuric acid. This was repeated several times, and then the openings washed out with dilute hydrogen-dioxide. No cotton was left in the openings, as they were amply patulous.

I did not see the case again for five days; patient reported feeling nearly well, with the exception of some remaining soreness in the face; swelling nearly gone. Adjusting the rubber dam to the tooth, all dressing was removed and the cavity and canals thoroughly dried with absolute alcohol. Finding no odor or moisture, the whole interior of canals was wiped with formalin and then dried with alcohol again. Using gold wire, 22 carat and 28 gauge, I taper it down at the end with a fine file and try it into the canal until it goes easily to the end, then cut it off, just long enough to come up into the main cavity. In this way I prepare a wire for each canal, and lay them on my operating-table in such order that I may easily distinguish them. Now, with oxychloride of zinc cement, mixed quite thin, I fill each canal, using a gold broach to introduce the cement. I then introduce each gold wire into its respective canal, pushing them home to the end. Over all I place a little of the oxychloride mixed a little stiffer, and allow it to set.

This constitutes what I consider to be the most thorough method of filling root-canals, avoiding all boring or enlargement of canals, which I consider very dangerous. I saw my patient four days afterwards, and found the condition of the face much improved. Some of the openings closed, others partially so, no swelling and no soreness.

I completed the filling of the tooth, and told the patient that, unless some aggravation set in, he need not come for a week. I did not see him for two weeks, when all conditions seemed normal. Slightly reddish spots were the only vestige of the openings. I think the cure was complete, as all symptoms of the trouble had disappeared.

*President Draper.*—Dr. Davis's paper is before you for discussion. Is there anything to be said on this matter?

*Dr. Maxfield.*—Inasmuch as the room is getting close and dark, I would suggest that the reading of the two remaining papers be deferred until to-morrow morning.

Motion seconded and carried.

### *Afternoon Session.*

President Draper called meeting to order, and then followed Dr. Palmer's paper.

For Dr. Palmer's paper, see page 84.

*President Draper.*—Dr. Palmer's paper is now open for discussion, and we will listen to a few remarks by Dr. C. E. Stockwell, of Springfield, who has consented to open the discussion. As Dr. Stockwell is not feeling well, it would be a favor to him if you would move up closer, so he would have to exert himself less.

*Dr. C. T. Stockwell.*—It was in January, I believe, that Dr.

Williams gave the paper in New York which our friend, Dr. Palmer, was challenged to answer and which he has reviewed so ably to-night. I hope that every member present read that paper very carefully. If he did not do so, he may be surprised to learn that about one-third of that paper was given to a discussion of theories which he has supported the last twenty-five years or so. In that discussion, as Dr. Palmer has stated, was a challenge to me. This occurred in New York, and, unless I am mistaken, believed to have been, as I think it was intended, to mark an epoch in the history of dentistry upon the intellectual state of men. Because of these facts, I am exceedingly glad that Boston and the Massachusetts Dental Society have offered Dr. Palmer an opportunity and the vehicle for meeting that challenge. Another thing, I feel like apologizing to you gentlemen for occupying ten minutes or so of your time. If I take you out, or invite you to follow me out, towards the outermost rim of what I apprehend to be physical science at present, and you think I have taken you further from what properly belongs to the science of dentistry, I shall ask your forgiveness, and promise you that I never will do so again.

Quite unexpectedly I found, only a few days since, my name upon the programme as the one designated "to lay his mind alongside" this paper of Dr. Palmer's "and try to conquer it." In other words, it was announced, without my knowledge or consent, that I was to open the discussion. This selection on the part of our committee would be regarded as a personal compliment of no little value did I consider myself competent to discuss such a paper. But the consciousness that I belong to that class which you have heard denominated as "book-made men" rather than to the few who form their conclusions solely from a direct study of nature,—this consciousness, I say, makes me hesitate about venturing upon any extended discussion.

Some things, however, are pretty clear to even "book-men," and, in my opinion, they possess certain advantages over the merely scientific quarryman who delves for the facts of nature. His sweep of vision may be wider. His horizon is likely to be broader. He is less imprisoned in a partial and sometimes a partisan logic relating to a class of facts. The "book-made man," such as Herbert Spencer, for instance, sees, and is chiefly interested in, *the relations* that one fact sustains to another fact; or, to put it in its broadest senses, the relation that one fact sustains to all obtainable facts as well as the relations sustained by all the facts to the single fact.

And so I say, without fear of contradiction, that the highest knowledge is the knowledge, not of facts alone, but of *relations*. True it is that synthesis is based upon analysis. But a broad synthesis must be based upon no partial or special line of analysis. True it also is that the world has had but few men who were at the same time broadly possessed of these two qualities. All real workers in physical science admit this fact. I have personally heard such men say to these berated book-men: "We will give you the facts. You shall build the building. We are the quarrymen; you the architects."

In the very nature of things this must be so. Life is not long enough for any one man to become a genius in both spheres of human activity. The theories, the creeds, the synthetic philosophies, in short, must be constructed from the work of the great army of those who are, and well may be, content to delve in the great mine of nature and bring forth the golden facts from her infinite storehouse of divine beneficence. And while doing this, no real, sane, scientific worker will waste any energy in trying to belittle the "book-made man" who, revolving in his mind the recorded facts of nature, tries to discern RELATIONS.

What are the books but a printed record of the facts which scientific workers, like Dr. Williams, for instance, have given to the world? Does he advise us to forego the pleasure and profit we derive in reading his own papers? On the other hand, he announces in this very paper which our essayist this evening has reviewed, that if he could know that every student in our dental colleges would read this paper he would be content. What are the one hundred thousand or more of original observations from nature made by or under the direction of Mr. Edison to which Dr. Williams calls attention? Would not these observations make a book worthy of some attention by even other scientific workers? What, after all, is Nature other than a great book,—*the* book out of which flow the issues of life, and to the reading and interpretation of which the entire scientific world is summoned?

Taking the matter, however, in the narrower sense in which I presume the argument to be intended, I will admit that both classes of men possess certain advantages over the other. The man who "lays his mind alongside a physical fact, and conquers it," sees more in that fact than he can report, or put into a book. On the other hand, because of his devotion to a special line, or, at most, to special lines of work, he is apt to be less fitted in the way of broad interpretations of even his own observed facts. The real book-made man has here an advantage, and in so far as his vision takes within its scope all the facts of nature, so far are his interpretations nearer the ultimate truth.

This much I desire to add to what Dr. Palmer has said in answer to the spirit, if not the exact letter, of parts, at least, of Dr. Williams's paper.

Without attempting to discuss Dr. Palmer's paper (for it seems to me that this is a paper which the audience needs to have time to carefully study and digest before it can be profitably discussed), I may, perhaps, be permitted to call your attention to a few of its salient points; and, first of all, I wish to bring out clearly the fact that Dr. Palmer does not appear here to controvert the scientific conclusions of Drs. Miller, Black, and Williams. Let the point at issue be fully understood. He admits all that these gentlemen claim for their *investigations.* He admits that, as far as they go, they demonstrate, to the acceptance of the scientific world, the accuracy of their work. But Dr. Palmer takes exceptions to the claim that all knowledge of the causes of dental caries is limited to the realm of purely physical demonstration. He does not admit that the last word has been said, and that the subject is closed. He asks them, and us, to go further; and he well says that "the limit to knowledge is bounded by capacity of comprehension." Science itself is in evolution, as he says; and science cannot be defined in terms of physical expression alone. Huxley, the authority which Dr. Williams quotes so freely, says of science, "To my mind, whatever doctrine professes to be the result of the application of the accepted rules of inductive and deductive logic to its subject-matter, and accepts, within the limits which it sets to itself, *the supremacy of reason,* is science." Professor DuBois's (Yale Scientific School) definition is of a still deeper insight. He asks: "May we not define all science as the verification of the ideal in nature?" A more common definition is as follows: "Science is that interpretation of phenomena which best explains all the facts of consciousness."

It seems to me, therefore, that Dr. Palmer's claim, that science cannot be limited to the realm of the purely physical world, is well taken. If it were not so, science would justly be chargeable with gross materialism, something which it long since outgrew, even if the charge ever had any substantial foundation. It is but a very superficial knowledge that to-day brings the charge of materialism against science; and if I were called upon to point out the greatest of all

"influences that retard evolution in dental science," it would be, in my opinion, the narrow, but common, conception of what constitutes science.

True it is, as Dr. Palmer says, "science relates to the *inner life* of teeth as well as to their environment."

Remember, also, that the essayist is dealing with developing or young teeth,—teeth that are still in the active process of building. And when he says that "the functions of the pulp are such as to intelligently direct the construction of the tooth," he touches upon one of the most tremendous and far-reaching deductions that is beginning to be conceded by the world's greatest naturalists, viz., That mind is a correlative aspect of matter; that life and consciousness are practically synonymous terms; that every atom in the universe is alive, no matter in what particular form or combination it may be found; that every particle of matter possesses not only consciousness, but the elements of choice.

The great German naturalist and biologist, Haeckel, came practically to this conclusion years ago. The late lamented Professor Cope, one of America's greatest scholars and scientists, a man as marvellous in his power of synthesis as in his almost, if not quite, unequalled power of analysis, held, with Haeckel, that not only are all the atoms of matter endowed with life and consciousness, but he went a step further, and held that life and consciousness *precede* organism; that life consciously builds its own body or bodily form. Note, also, as apropos to this matter, his definition of life: "I think it possible to show," he says, "that the true definition of life is: Energy directed by sensibility, or a mechanism which was originated under the direction of sensibility." The physicists, also, such as your own Professor Dolbear, of Tufts College, tell us that everything we eat, drink, or breathe is alive, and that these give us life because they are alive, not dead. Our own governmental agriculturists, in their published reports, tell us that the very soil of our farms and our streets is organic; that, instead of being dead, inert matter, as so commonly believed, it is, rather, a mass of living organisms. Chemists, too, in some of their later works, are placing a different emphasis on the words "chemical affinity" than formerly. In some cases that have come to my notice, the most conservative of them are now speaking of the term "affinity" as more or less analogous to the term "choice." And Powell, another of our leading American scientists, in his late book on "Truth and Error, or the Science of Intellection," as one of the fundamental, if not the leading, proposition of his thesis, makes consciousness one of the primary attributes of the ultimate particles of matter, holding that they not only possess consciousness, but choice as well. These, and many others, may be cited as establishing the fact that life and consciousness are universal and eternal, varying in their phenomena only as organization varies. And it is because of the facts presented by these and like scientific men of our own day and generation that these conclusions are being accepted by the more advanced minds in the scientific world.

And yet, while our modern scientific literature is permeated with such ideas and hypotheses, some of our would-be dental scientific authorities are telling us that enamel is lifeless, because, forsooth, under their microscopes they fail to find organized nerve-fibrils. They forget, it must be, that "there is no longer any use of denying that science has bridged the gulf between the organic and the inorganic," to quote the language of one of our conservative New England college professors of science. Dead matter! There is nothing dead in all the universe but death. Does such a statement need to be substantiated by a physical demonstration in some of our laboratories in order to be acceptable to the rational mind? Not so. It needs, rather, a receptive mind towards the marvellous array of facts that our psychologists, biologists, physicists, and chemists are bringing out in these eventful years, and the perception that can take in, to some extent at least, the *relation* of these facts as correlated phenomena. Mind, not "matter," is now seen to be the dominating factor in organic evolution,—that is, mind and matter are two phases of one and the same thing. It is mind *in* matter, not mind *and* matter,—two separate and distinct entities.

I have dwelt upon this point somewhat for the reason that it seems to me a most important and practical point. From this point of view, while not forgetting in our daily work the conditions of environment, we shall have a deepened and broadened outlook relative to the inner life and functions of these organs that come under our professional care.

There is one point in the paper which seems to me open to adverse criticism. For instance, when the author says that "Force is the life of matter, and that force is electricity," I should not only substitute the word energy for "force," but I should say, rather, that energy is the *manifestation* of life in matter, and that electricity is one of the various and multiple *forms* of energy.

All matter exhibits some form of energy, and it is because of this fact, or partly so, that all matter is believed to possess life. Life itself is a form of energy, consciousness is a form of energy, mind is a form of energy, and so all forms of matter and all forms of energy come properly within the scope and meaning of the term "vital," as it is commonly used. The origin also of this vital or life-energy, I might say, is now being traced back to some superphysical source embodied in the universal ether. I say superphysical, because, according to our physicists of to-day, ether-energy is an *endowment*, and atomic energy is an *embodiment;* and it is seen clearly now that some form of energy radically different from any known to physical science is required to embody ether-energy in the forms of atoms.

That is to say, the day has come when science, in its analysis of matter, traces it back to an immaterial substance, the ether, and all forms of physical energy back to a unit-energy,—an energy something other, something else, something radically different from any form of energy known to physical science. In short, it is traced back not to the supernatural, but to a superphysical form of energy, manifesting itself in the universal ether as *universal Mind.* Dr. Palmer calls it God. Names are of little account. The great fact is what we want to bring into consciousness. To say, however, of anything, or of any phenomenon, "God did it," explains nothing, as has been well said by a well-known scientific man. I suspect that man is encouraged to try, at least, to find out how "God did it," in order that he (man) may acquire a closer likeness to the "image" Dr. Palmer speaks of. For the image is of the nature of the mental, or psychical, rather than of any physical form. And I do not suppose Dr. Palmer or any other thoughtful man will question the truthfulness of the remark. It should not be understood, however, as Dr. Williams apparently would have us understand, that science has made no progress since the days of Tyndall, some twenty years ago, in accounting for the sources of energy, be they physical or so-called "vital." I dislike the term vital as much as he seems to dislike it. The term life-energy suits me better. For all energy is, at last, one, and one in origin.

Tyndall's statement, therefore, though good as far as it went, is not sufficiently modern to be wisely quoted as utterly disposing of the class for which it was used. And so I should say that Dr. Palmer's position, as set forth in his paper, is nearer the view of modern science than that of his critic, Dr. Williams.

If the foregoing should seem to be a vague glimpse towards the outermost rim of speculative science, it is, I can assure you, not a glimpse too far away to be of use to us in our daily work. Let the young men here this evening especially make a note of this remark. It is just here where the world's best physicists are to-day, carefully and with hushed voices, reverently feeling their way. And they are agreed, so we are told, that before the first speck of ether was whirled into a vortex-ring, constituting the first atom of matter, Mind, Consciousness, Will, *was*. But with the apparition of the first atom *physical* evolution began. This is where "the supremacy of reason," which Huxley denominates science, carries us to-day.

I do not know enough about the science of electricity in detail to warrant any attempt to discuss much that Dr. Palmer has presented in this paper. I will, therefore, cheerfully leave that part to others, with the simple remark that I am intensely interested in this statement of the matter as it presents itself in its various bearings to his mind after so many years of faithful, earnest, devoted, careful, and self-sacrificing work which he has given to it. There is much in the paper of great practical importance. Of this I am sure, from the fact that I have availed myself of some of these suggestions in my own practice, and have watched results. I allude here to other occasions when he has brought out some of these points and enriched the profession from his great store of experience and knowledge. And as I read the paper in advance, with reference to my duty this evening, I was again deeply impressed with the conviction that the profession owes to Dr. Palmer a great debt of gratitude.

*Dr. R. R. Andrews* (Cambridge, Mass.).—I had made up my mind to say something in relation to Dr. Palmer's and Dr. Stockwell's papers. But in view of the lateness of the hour, it will be manifestly unfair and unjust to our essayist from New York, so I move that, in place of further discussion of the preceding papers, we listen to the paper from Dr. Bogue.

*Dr. E. A. Bogue.*—I do not think it would be fair to the Society not to have further discussion of Dr. Palmer's paper.

*President Draper.*—I think the Society is in accord with Dr. Andrews's suggestion. We will put it to a vote.

On putting the question, it was the sense of the meeting that Dr. Bogue proceed.

